# Predictors of the effects of treatment for shoulder pain: protocol of an individual participant data meta-analysis

**DOI:** 10.1186/s41512-019-0061-x

**Published:** 2019-08-08

**Authors:** Danielle A. van der Windt, Danielle L. Burke, Opeyemi Babatunde, Miriam Hattle, Cliona McRobert, Chris Littlewood, Gwenllian Wynne-Jones, Linda Chesterton, Geert J. M. G. van der Heijden, Jan C. Winters, Daniel I. Rhon, Kim Bennell, Edward Roddy, Carl Heneghan, David Beard, Jonathan L. Rees, Richard D. Riley

**Affiliations:** 1 Institute for Primary Care and Health Sciences, Arthritis Research UK Primary Care Centre, Centre for Prognosis Research, Keele University, Keele, UK; 2 School of Health Sciences, University of Liverpool, Liverpool, UK; 3 Department of Social Dentistry, Academic Center for Dentistry Amsterdam, University of Amsterdam and Vrije Universiteit Amsterdam, Amsterdam, The Netherlands; 4 Department of General Practice and Elderly Care Medicine, University Medical Centre Groningen, Groningen, The Netherlands; 5 San Antonio Military Medical Center, Fort Sam Houston, San Antonio, TX USA; 6 Centre for Health, Exercise and Sports Medicine, Department of Physiotherapy, University of Melbourne, Melbourne, Australia; 7 Haywood Academic Rheumatology Centre, Staffordshire and Stoke-on-Trent Partnership Trust, Stoke-on-Trent, UK; 8 Nuffield Department of Primary Care and Health Science, University of Oxford, Oxford, UK; 9 Nuffield Department of Orthopaedics, Rheumatology and Musculoskeletal Sciences (NDORMS), University of Oxford, Oxford, UK

**Keywords:** Shoulder conditions, Pain, Disability, Individual participant data meta-analysis, Predictors of treatment effect

## Abstract

**Background:**

Shoulder pain is one of the most common presentations of musculoskeletal pain with a 1-month population prevalence of between 7 and 26%. The overall prognosis of shoulder pain is highly variable with 40% of patients reporting persistent pain 1 year after consulting their primary care clinician. Despite evidence for prognostic value of a range of patient and disease characteristics, it is not clear whether these factors also predict (moderate) the effect of specific treatments (such as corticosteroid injection, exercise, or surgery).

**Objectives:**

This study aims to identify predictors of treatment effect (i.e. treatment moderators or effect modifiers) by investigating the association between a number of pre-defined individual-level factors and the effects of commonly used treatments on shoulder pain and disability outcomes.

**Methods:**

This will be a meta-analysis using individual participant data (IPD). Eligible trials investigating the effectiveness of advice and analgesics, corticosteroid injection, physiotherapy-led exercise, psychological interventions, and/or surgical treatment in patients with shoulder conditions will be identified from systematic reviews and an updated systematic search for trials, and risk of bias will be assessed. Authors of all eligible trials will be approached for data sharing. Outcomes measured will be shoulder pain and disability, and our previous work has identified candidate predictors. The main analysis will be conducted using hierarchical one-stage IPD meta-analysis models, examining the effect of treatment-predictor interaction on outcome for each of the candidate predictors and describing relevant subgroup effects where significant interaction effects are detected. Random effects will be used to account for clustering and heterogeneity. Sensitivity analyses will be based on (i) exclusion of trials at high risk of bias, (ii) use of restricted cubic splines to model potential non-linear associations for candidate predictors measured on a continuous scale, and (iii) the use of a two-stage IPD meta-analysis framework.

**Discussion:**

Our study will collate, appraise, and synthesise IPD from multiple studies to examine potential predictors of treatment effect in order to assess the potential for better and more efficient targeting of specific treatments for individuals with shoulder pain.

**Systematic review registration:**

PROSPERO CRD42018088298

## Background

Musculoskeletal conditions are globally among the leading causes of years lived with disability [1]. Shoulder pain is one of the most common presentations with a 1-month population prevalence of between 7 and 26% [2]. Annually, about 4% of adults will visit their general practitioner (GP) for shoulder pain [3, 4], resulting in approximately 1.5 million consultations in England. The total annual costs of shoulder pain to society have been estimated at £100 million for the UK [5]. Mean annual costs per patient have been estimated at £3500 (Sweden) [6] and £8500 (the USA) [7] per patient, with costs of surgical interventions and work absence contributing most to this estimate.

Systematic reviews (e.g. [8–14]) as well as recently published trials (e.g. [15–18]) consistently show moderate short-term effects of primary care interventions such as corticosteroid injection and exercise, but a lack of evidence for long-term (> 6 months) benefit. The rates of surgery in the UK increased by more than sevenfold (750%) for subacromial decompression alone from 5.2/100,000 in 2001–2002 to 40.2/100,000 in 2009–2010 [19], but there is increasing evidence that some surgical shoulder interventions are no more effective than non-surgical treatments or placebo surgery [20–25]. This demonstrates an increase in the use of healthcare resources, without evidence of improvement in long-term patient outcomes, and little guidance to support optimal treatment and referral decisions. The implementation of more promising or better-targeted treatment for shoulder pain is hampered by a lack of high-quality evidence for the predictive value and clinical utility of diagnostic and prognostic information.

Clinicians use information from the patient’s clinical history and physical examination to assess the severity and possible origin of the problem (rotator cuff tears or tendinopathy, subacromial bursitis, adhesive capsulitis/frozen shoulder, acromioclavicular conditions, glenohumeral osteoarthritis), although diagnostic accuracy is limited [26–28]. Qualitative research shows inconsistency in the labelling of shoulder conditions and little impact of diagnostic labels on treatment decisions [29]. Based on their clinical assessment, clinicians decide which type of treatment (e.g. advice and pain relief only; exercise with or without mobilisation; corticosteroid injection; referral for a specialist (orthopaedic) opinion) is most suitable for their patient. There is little evidence, however, to support such decisions. The uncertainties clinicians experience in the management of shoulder pain and the different strategies they use to deal with these have been confirmed in a recent interview study among Dutch GPs [30] and in surveys conducted in Australia [31] and the UK [32].

The overall prognosis of shoulder pain is highly variable with 40–50% of patients reporting persistent pain 6–12 months after consulting their primary care clinician [33–36]. In addition to diagnostic information obtained from the clinical assessment, the long-term prognosis can be influenced by a range of factors, including age, educational level, symptom duration, previous episodes, disability scores, and multisite pain [35–40]. The prognostic value of psychological and social factors is less clear, but evidence suggests that fear-avoidance beliefs, pain catastrophizing, depressive symptoms, low pain self-efficacy, and aspects of the psychosocial work environment (high job strain, low supervisor/co-worker support) are associated with poor long-term outcome [36, 38, 39, 41–46], particularly in those with chronic or recurrent shoulder pain [47, 48].

Qualitative research in patients with shoulder pain has emphasised the anxiety patients experience due to uncertainty about prognosis and treatment. Patients highlighted the lack of clear, written information about the condition and expressed a wish for faster, better-defined treatment choices [49]. This was confirmed by results of a recently completed James Lind Alliance Priority Setting Partnership, with one of the top 10 research priorities being ‘How can we ensure that patients see the right clinicians promptly and correctly, and does this lead to better outcomes?’ [50] Despite evidence for prognostic value of a range of patient and disease characteristics, it is not clear whether some of these factors may also predict the effectiveness of specific treatments (such as corticosteroid injection, exercise, or surgery) in an individual or subgroups of similar individuals, i.e. modify or moderate the effect of treatment, and may inform decisions regarding the type of treatment likely to be most effective for specific (subgroups of) patients [51]. There is a clear need for research investigating individual-level predictors of treatment effect in order to assess the possibility of better and more efficient targeting of shoulder pain treatments, potentially improving patient outcomes.

### Study objectives

Investigating treatment effect moderation requires data from randomised controlled trials in order to estimate candidate treatment-predictor interactions and describe subgroup effects. A single randomised trial is generally not powered to detect genuine individual-level predictors. To address this, we will collate and synthesise individual participant data (IPD) from multiple trials to increase the power to identify genuine predictors of treatment effect. Our aim is to determine moderation of the effects of four types of treatments that are most commonly used in the primary care management of painful shoulder conditions and where evidence regarding predictors of treatment effect would inform decisions regarding their optimal use: (a) corticosteroid injection, (b) physiotherapy-led exercise, (c) psychological treatments, and (d) surgical interventions on shoulder pain and disability outcomes by a limited number of a priori defined individual-level candidate predictors (patient characteristics). The IPD meta-analysis will be conducted in three steps with the following objectives:

1. To estimate the overall effects of each of these four treatments separately, when compared to a control intervention (no treatment, advice and analgesics only, or sham)
2. To estimate the relative effects when these interventions are directly compared against each other in pragmatic randomised trials
3. Within each of these comparisons separately, to estimate treatment-predictor interactions for a number of a priori defined patient or disease characteristics and describe relevant subgroup effects when interactions are detected

The results of the IPD meta-analysis will be used to define profiles of patients with shoulder pain likely to respond well to each of the treatments listed above. As part of a larger programme of research, the IPD meta-analysis will form the basis of the design of a treatment decision tool for patients consulting with shoulder pain in primary care, to be used as part of a stratified care intervention and tested against usual care for shoulder pain in a large pragmatic randomised controlled trial.

## Methods

An a priori protocol was established for this IPD meta-analysis and registered with the international prospective register of systematic reviews: PROSPERO (www.crd.york.ac.uk/prospero/), number CRD42018088298.

### Eligibility criteria (see also Table 1)

#### Design and setting

IPD will be included from randomised clinical trials conducted in the community, primary healthcare, or secondary healthcare settings. No language restrictions will be used.

**Table 1:** Eligibility criteria for including trials in the IPD meta-analysis

	Include	Exclude
Design	Randomised clinical trials, including individual and cluster-randomised trials, no language restrictions	Any other non-randomised design
Setting	Any healthcare setting	Preventative intervention studies (e.g. in workplace settings)
Population	▪ Adults (18 years and older) ▪ General or non-specified shoulder pain or diagnosed with (i) subacromial condition (rotator cuff tendinopathy, rotator cuff tear, subacromial bursitis), (ii) frozen shoulder/adhesive capsulitis, (iii) glenohumeral osteoarthritis, (iv) shoulder instability.	▪ Acute trauma (fractures, traumatic dislocations) ▪ Inflammatory arthritis (rheumatoid arthritis, polymyalgia rheumatica) ▪ Shoulder pain resulting from cervical radiculopathy ▪ Stroke-related shoulder pain
Interventions	▪ Corticosteroid injection ▪ Physiotherapy-led exercise (with or without manual therapy) ▪ Interventions addressing psychological factors (e.g. cognitive behavioural approaches, multimodal interventions) ▪ Surgical interventions	Other, less common treatment options, or treatments with very limited evidence of effectiveness
Control	▪ Advice and pain relief only ▪ Sham/placebo intervention ▪ Direct comparisons between the interventions listed above	▪ Comparisons with other interventions ▪ Comparisons of different dosages, types, or modes of delivery of the same intervention
Outcome measure	▪ Shoulder pain intensity (VAS, 0–10 NRS, validated shoulder-pain specific questionnaire) ▪ Shoulder function (NRS, VAS, validated shoulder disability questionnaire).	No baseline and follow-up data for either one of these outcomes
Candidate predictors	At least one of the following potential predictors in addition to baseline levels of the outcome measure (pain or disability): shoulder pain duration, sleep disturbance due to shoulder pain, presence of weakness, cause of shoulder pain (injury, or overuse due to work/hobbies), co-existing neck pain, psychosocial complexity (fear-avoidance, catastrophizing, anxiety, depression, other), positive expectations or preferences regarding treatment, presence of comorbidities.	
Length of follow-up	Follow-up assessment at least 4 weeks after randomisation	Trials including only very short-term follow-up (e.g. after a single intervention session)
Sample size	Minimum sample size at randomisation, 30 per intervention arm	

#### Study population

The IPD meta-analysis will include trials conducted in adult patients with general or non-specified shoulder pain or diagnosed with subacromial conditions including rotator cuff tears, rotator cuff tendinopathy, subacromial impingement, or subacromial bursitis; (ii) frozen shoulder or adhesive capsulitis; (iii) glenohumeral osteoarthritis; and (iv) shoulder instability. The IPD meta-analysis will not include trials focusing on acute trauma (fractures, traumatic dislocations), inflammatory arthritis, shoulder pain resulting from cervical radiculopathy, or stroke-related shoulder pain.

#### Interventions

The IPD meta-analysis will focus on interventions that are commonly used in the primary care management of shoulder pain in the UK. Trials will be included that investigate the effectiveness of the following:

- Corticosteroid injection versus injection of an anaesthetic only or compared with control (advice and analgesics or no additional treatment)
- Physiotherapy-led exercise therapy (with/without manual therapy) compared with control (advice and analgesics or no additional treatment)
- Corticosteroid injection compared with physiotherapy-led exercise therapy (with/without manual therapy)

Primary care clinicians may also consider referral of patients with shoulder pain to secondary care services, offering more extensive treatments for shoulder pain. We will therefore also include trials investigating the effectiveness of the following:

- Interventions incorporating assessment and management of psychological risk factors, such as cognitive-behavioural approaches, or multimodal treatment programmes compared with exercise, injection, and/or control (advice and analgesics or no additional treatment)
- Surgical treatment compared with non-surgical treatment (exercise, injection) and/or control (advice and analgesics or sham surgery)

#### Outcome measures

We will include trials that have used (i) a measure of shoulder pain (e.g. visual analog scale (VAS), 0–10 NRS, validated shoulder-pain specific questionnaires) and/or (ii) a measure of shoulder pain related disability (NRS, VAS, validated shoulder disability questionnaires).

#### Length of follow-up

Trials will be included that have a follow-up assessment at least 4 weeks after randomisation. Outcomes will be analysed for up to 24 months after randomisation.

#### Sample size

In order to reduce the risk of small-study bias, we will only include trials with a sample size of at least 30 participants per treatment arm at the time of randomisation. This cut-point is arbitrary, but will ensure we obtain data for the largest trials. Furthermore, excluding the smallest trials will allow a more efficient approach to collecting data for this IPD meta-analysis, which is otherwise known to be very time-consuming [52]. Smallest trials are also more prone to baseline imbalance (by chance), and thus their exclusion will reduce the risk of identifying spurious treatment effects and treatment-predictor interactions.

#### Candidate predictors

Developmental work has been conducted which has generated a shortlist of candidate predictors to be analysed in this IPD meta-analysis. A systematic review of randomised clinical trials identified 21 trials that investigated or suggested candidate predictors of the effects of treatment with corticosteroid injection, strengthening exercise and/or mobilisation, or advice and pain relief [53]. Only 7 trials included a moderation analysis or subgroup analysis, whereas 14 trials made untested suggestions of effect modification. The results of the systematic review informed a series of workshops with clinicians, who proposed profiles of patients most likely to respond to these treatments, and prioritised a list of 12 potential predictors. These 12 candidate predictors formed the basis of an international choice-based conjoint analysis survey [53], and most of these will be tested in the proposed IPD meta-analysis: baseline pain severity, baseline disability, sleep disturbance due to shoulder pain, presence of instability or weakness, cause of shoulder pain (injury or overuse due to work/hobbies), co-existing neck pain, psychosocial complexity (fear-avoidance, catastrophizing, anxiety, depression), positive expectations or preferences regarding treatment, and presence of comorbidities. Additionally, the author team suggested shoulder pain duration as an important candidate predictor of treatment effect.

### Patient and public involvement

The perspectives of patients and clinicians involved in the management of shoulder pain be included in the design and reporting of the IPD meta-analysis, by involving an advisory group of people with experience of living with shoulder pain, and a clinical advisory group including physiotherapists, rheumatologists, GPs, and orthopaedic surgeons. Patient representatives will be invited from Keele University Research User Group. The advisory groups will be consulted regarding (i) candidate predictors and outcomes, (ii) interpretation and importance of findings to patients and clinicians, and (iii) how best to present the findings to the general public, patients, and clinicians.

### Searching and selection

Potentially eligible trials were identified through existing relevant systematic reviews and an updated search of individual trials published after the search dates of these reviews (up until May 2018). Updated searches of electronic databases (restricted to randomised controlled trials) included MEDLINE, EMBASE, Cochrane Central Register of Controlled Trials (CENTRAL), PEDro, WHO ICTRP, and ClinicalTrials.gov. Table 2 presents the searches for MEDLINE (through Ovid) as an example; search terms were adapted for other databases. Citations will be imported into Covidence for screening and selection.

**Table 2:** Searches to identify systematic reviews and potentially eligible trials (MEDLINE via Ovid)

Systematic reviews	Randomised clinical trials
1. exp Shoulder Joint/	1. exp PAIN/
2. shoulder.ti,ab.	2. exp Shoulder Joint/
3. “rotator cuff”.ti,ab.	3. 1 and 2
4. glenohumeral.ti,ab.	4. Shoulder Pain/
5. subacromia$.ti,ab.	5. Shoulder Impingement Syndrome/
6. or/1-6	6. exp Bursitis/
7. Pain/ or pain.ti,ab.	7. rotator cuff/
8. 6 and 7	8. ((shoulder or subacromial or glenohumeral or rotator cuff) adj3 (instability or bursitis or frozen or impingement or tendinitis or tendonitis or pain$ or osteoarthr$ or periarthriti$)).ti,ab.
9. exp Shoulder Pain/	9. “adhesive capsulitis”.ti,ab.
10. “adhesive capsulitis”.ti,ab.	10. or/3-9
11. Shoulder Impingement Syndrome/	11. exp Adrenal Cortex Hormones/
12. or/8-11	12. (steroid or corticosteroid).ti,ab.
13. Meta-Analysis as Topic/	13. 11 or 12
14. meta analy$.tw.	14. inject$.ti,ab.
15. metaanaly$.tw.	15. 13 and 14
16. Meta-Analysis/	16. exp Exercise/ or exp Exercise Therapy/
17. (systematic adj (review$1 or overview$1)).tw.	17. exercise$.ti,ab.
18. exp Review Literature as Topic/	18. 16 or 17
19. OR/13-18	19. exp Musculoskeletal Manipulations/
20. cochrane.ab.	20. (“manual therapy” or manipulation$ or mobilisation$).ti,ab.
21. embase.ab.	21. (chiropract$ or osteopath$).ti,ab.
22. (psychlit or psyclit).ab.	22. 19 or 20 or 21
23. (psychinfo or psycinfo).ab.	23. exp adaptation, psychological/ or exp behavior/ or exp psychology, social/
24. (cinahl or cinhal).ab.	24. (psychosocial or psycholog$).ti,ab.
25. science citation index.ab.	25. behavio$.ti,ab.
26. bids.ab.	26. 23 or 24 or 25
27. cancerlit.ab.	27. (surgery or surgical or operat$).ti,ab.
28. “web of science”.ab.	28. exp orthopedic procedures/ or orthopedics/
29. or/20-28	29. 27 or 28
30. reference list$.ab.	30. (physio$ or physical therap$).ti,ab.
31. bibliograph$.ab.	31. exp Physical Therapy Modalities/
32. hand-search$.ab.	32. 30 or 31
33. relevant journals.ab.	33. 15 or 18 or 22 or 26 or 29 or 32
34. manual search$.ab.	34. 10 and 33
35. or 30-34	35. random$.ti,ab.
36. selection criteria.ab.	36. factorial$.ti,ab.
37. data extraction.ab.	37. crossover$.ti,ab.
38. 36 or 37	38. cross over$.ti,ab.
39. Review/	39. placebo$.ti,ab.
40. 66 and 67	40. (doub$ adj blind$).ti,ab.
41. Comment/	41. (sing$ adj blind$).ti,ab.
42. Letter/	42. assign$.ti,ab.
43. Editorial/	43. allocat$.ti,ab.
44. animal/	44. volunteer$.ti,ab.
45. human/	45. double-blind procedure/
46. 44 not (44 and 45)	46. crossover-procedure/
47. 41 or 42 or 43 or 46	47. randomized controlled trial/
48. 19 or 29 or 35 or 40	48. single-blind procedure/
49. 48 not 47	49. or/35-48
50. 12 and 49	50. 34 and 49

Two reviewers will screen titles and abstracts of trials identified from these searches against the eligibility criteria described above, excluding trials that clearly do not meet the eligibility criteria. Subsequently, full texts of trial reports will be retrieved and assessed for eligibility, again by two reviewers independently. Disagreements will be resolved through discussion or by third reviewer adjudication. Trial authors will be contacted if there are queries regarding eligibility.

### Extraction of aggregate data

For each included trial, details on study design (randomisation and allocation procedure), study setting, sample size, baseline characteristics of the study sample (age, gender, diagnosis, duration and history of shoulder pain), details of interventions (type of treatment, duration, frequency, dose, co-interventions), comparator (advice, analgesics, other), candidate predictors, and outcome assessment (type of outcome measure, timing of follow-up, numbers lost to follow-up) will be extracted into tables. Two reviewers will independently extract outcome data on self-reported pain and disability at time points nearest to 6 weeks (short-term), 3–6 months (medium-term), and ≥ 12 months (long-term).

### Risk of bias assessment

All trials identified from our searches will be assessed using the Cochrane Risk of Bias tool, regardless of whether they supply their IPD or not. Two researchers will independently grade risk of bias (unclear, high, or low risk of bias) based on sequence generation, allocation concealment, blinding of outcome assessor, incomplete outcome data, and selective outcome reporting. Although our aim is to obtain IPD from all studies, we recognise some teams may decline or no longer have access to data [54]. In this situation, we will also compare the risk of bias for those studies that do and do not provide their IPD. Studies providing their IPD will have a more thorough examination of their risk of bias, as aspects of design and conduct can be checked with the trial team, and the IPD allows an examination of some aspects to a greater detail (e.g. baseline balance and missing information) than possible with the study publication alone [55].

### Data transfer and quality assurance of individual participant data

Principal investigators (PIs) or corresponding authors of identified trials will be contacted to inform them about the study and ask if they would be willing to share IPD. If there is no response, the institutes (e.g. head of department or research group) in which the trials have been performed will be contacted.

Those who express an interest in collaboration will be invited to read the protocol and requirements for sharing IPD will be discussed. The data custodian/representative of the institute who owns the data will then be invited to sign a data sharing agreement, which specifies the data requested, obligations, ownership of data, terms, authorship, and publications.

IPD requested from each trial will include the following:

- Baseline characteristics of participants (sociodemographic variables, characteristics of the shoulder condition, data regarding all candidate moderating variables)
- Randomisation code indicating random treatment allocation, any variables indicating non-adherence to protocol (protocol deviations), and duration and number of treatment sessions if these vary between individual participants
- Baseline and follow-up data regarding pain and disability outcomes at each time-point, where relevant including final scores for multi-item scales, and subscale scores for multidimensional scales (e.g. pain and function for SPADI). If available, EQ-5D will be requested as it contains items for pain and disability and may be used if trials have not included specific scales for pain or disability.

Trial authors will be asked to share relevant data regarding candidate predictors and outcomes regardless of whether such data has previously appeared in trial publications. The IPD received will not be used for any other research apart from that described in the data sharing agreement. All collaborators (one representative per trial) will be invited to be co-authors on manuscripts describing the principal analyses outlined in this protocol, subject to them meeting the International Committee of Medical Journal Editors criteria for authorship.

Collaborators will be invited to send their anonymised dataset as an encrypted file to the host institution, where this will be stored on a secure server. Prior to data transfer, there will be a process to verify that the person receiving the data is the accurate and intended recipient for the data. Any additional processes or requirements for data transfer stipulated by the collaborating institutions will be adhered to. Datasets will be accepted in any form, provided that all data are anonymised and variables and categories are adequately labelled in English. However, ideally, the format will be a two-dimensional spreadsheet with one participant per row and variables listed in columns and different time points on separate spreadsheets.

Datasets will be examined for missing and unusual values, with any issues resolved through communication with the original authors. We will attempt to reproduce the results included in each initial trial publication, including baseline characteristics and self-reported pain and disability at a time point nearest to 6 weeks, 3–6 months, and 12 months. Discrepancies or missing information will be discussed and clarified with original trial authors, where possible requesting analysis scripts. Following satisfactory data checking, each dataset will be converted to a common format and variables will be renamed in a consistent manner. The original scales of outcomes and covariate measurements that are reported will be utilised, where possible. To ensure compatibility across studies, when required, attempts will be made to convert variables to the same scale for all studies, by seeking to standardise the scale for outcomes and covariate measurements, for example, converting continuous outcomes to a standardised (e.g. N (0,1)) scale, by subtracting individual outcome values by the mean in the same study and then dividing by the standard deviation of values across individuals in the same study. Once each study data is cleaned and standardised, individual trial datasets will then be combined to form a new master dataset with a variable added to indicate the original trial.

### Approaches to dealing with missing participant-level data in trials

Participant-level missing data on candidate predictors and individual outcomes will be described. Under a ‘missing-at-random’ assumption, and where repeated outcome measures are available over time, individuals with partially missing outcome data (e.g. at some time-points) will be included in analyses (without imputation) using a longitudinal data (multi-level) modelling framework. Otherwise, they will be excluded, as this does not bias results for trials assuming the outcomes are missing at random [56]. If there is a considerable amount of missing baseline data for candidate predictors or covariates of interest (e.g. age, gender), this will be handled using multiple imputation, for those variables where a ‘missing-at-random’ assumption is deemed appropriate. Subsequently, for each meta-analysis, Rubin’s rule will be used to combine meta-analysis results across the imputed datasets [57].

### Analysis

#### General approach to the IPD meta-analysis

The IPD meta-analysis will be conducted in two steps: (1) estimating overall treatment effects on shoulder pain and disability outcomes for each of the treatment comparisons listed in objectives 1 and , and (2) estimating treatment-predictor interactions within each of the comparisons and separately for each of the candidate predictors (objective 3). We expect few trials will include data on all candidate predictors, and therefore, it is not our intention to develop a full prediction model that would consider all candidate predictors in a single model. Each of the candidate predictors will be separately tested for effect modification, using all trial datasets that have provided sufficient data on the treatment comparison, candidate predictor, and outcome measure.

For both steps, the IPD meta-analysis can be conducted using either a one-stage or two-stage approach [58]. Both approaches will be used in this IPD meta-analysis, with the two-stage approach being conducted as a sensitivity analysis. A two-stage approach is often preferred, as this approach automatically ensures that clustering of participants within trials is accounted for and aggregation bias can be avoided, which is more likely in a one-stage approach [59, 60]. This approach also allows the use of the Hartung-Knapp correction to inflate confidence intervals, accounting for the uncertainty of the estimated between-study variance [61]. However, the one-stage approach is likely to be more practical for modelling of non-linear associations of candidate predictors with outcome. Therefore, the analysis will be conducted using the one-stage approach (hierarchical model which will include both study-level and patient-level covariates in the same model), if possible using the Kenward-Roger method to inflate confidence intervals to account more fully for the uncertainty of variance estimates. Clustering will be accounted for by using a separate intercept per study and with separate residual variance and adjustment terms per study. Random treatment effects and treatment-predictor interaction terms will be included to allow for potential heterogeneity. Candidate predictors will be centred by their mean to avoid ecological bias [62]. A two-stage analysis will be performed as a sensitivity analysis to check whether conclusions are consistent.

#### Outcome measures

All analyses will be conducted for (i) severity of shoulder pain, measured using either a 0–10 numerical rating scale, 0–100 VAS, or pain scale (e.g. SPADI pain subscale), and (ii) level of shoulder function, measured using a validated shoulder disability questionnaire, with trials contributing to the analysis if they have provided sufficient data on any one of these measures.

#### IPD meta-analysis models

All analyses will be carried out using Stata 15 [63] or SAS 9.3 [64]. All analyses will be performed based on an intention-to-treat principle. All models will allow for random effects and will be estimated using restricted maximum likelihood (REML).

For both outcomes (shoulder pain and disability scores), the IPD meta-analysis will utilise a linear regression framework, where the final pain/disability score will be regressed against the baseline score, the treatment effect, the candidate predictor, and the interaction term. If studies use different pain or disability scales, these will be converted to a common scale if possible or otherwise estimates will be expressed as standardised mean difference. If standardised scales are required, the standardised final score will be regressed and the standardised baseline score adjusted for. For trials that contained multiple centres or a cluster-design, the hierarchical model will handle the clustering appropriately. Continuous candidate predictors will be entered as a linear term primarily, but sensitivity analysis will examine if non-linear trends are more plausible, using restricted cubic splines [65].

Adjustment for age and gender, as well as baseline pain/disability score, will be made in all analyses [66]. If there are repeated follow-up scores, the model will be adapted to include a repeated measures model where all follow-up times are jointly analysed, and the correlation among repeated measures from the same participants accounted for [67]. This approach can naturally handle missing follow-up scores for some patients, under a missing at random assumption. Treatment-predictor interactions may vary between time points, which will be an important criterion when interpreting statistically significant interaction terms (*p* < 0.10). Consistent evidence across multiple time points will add credence to it being a genuine (causal) predictor of treatment response [68]. Any model convergence problems will be reported clearly.

#### Data presentation

All summary and study-specific results will be presented in tables and via forest plots, with heterogeneity disseminated by *I*-squared, estimates of between-study variance and approximate 95% prediction intervals [69]. The entire process with be reported according to the PRISMA-IPD guidelines [70]. Overall treatment effects and treatment effects for each relevant subgroup (where significant treatment-predictor interactions will be identified) will be produced.

#### Small-study effects

Small-study effects refer to systematic differences in the effects from bigger and smaller studies and may occur due to many reasons, such as publication bias, heterogeneity, and availability bias (i.e. non-participation of trials toward the IPD meta-analysis). As mentioned, we aim to reduce the risk of small study bias by only including trials with a sample size of at least 30 participants per arm at the time of randomisation. However, in meta-analyses of 10 trials or more, small-study effects (which may highlight the potential for publication bias and availability bias) will be examined using contour-enhanced funnel plots and tests for asymmetry such as Egger’s test and Peter’s test [71]. In the presence of fewer than 10 trials, there is low power to detect small-study effects [72].

#### Sensitivity analyses

Sensitivity analyses will be carried out to assess the influence of important methodological factors and assess the robustness of the results of the main analysis. Sensitivity analyses will be based on (i) exclusion of trials studies at high risk of bias, (ii) use of restricted cubic splines to model potential non-linear associations for candidate predictors measured on a continuous scale, and (iii) the use of a two-stage IPD meta-analysis framework. For the latter, in the first stage, each trial providing IPD will be analysed separately to provide interaction estimates between each candidate predictor and treatment effect. Then, in the second stage, the interaction estimates will be synthesised using a random-effects meta-analysis, to produce a summary interaction estimate for each candidate predictor. REML will be used to estimate the random-effects meta-analysis models, with 95% confidence intervals for summary effects derived using the Hartung-Knapp approach to account for uncertainty in the estimated variance terms [73, 74]. Overall treatment effects and treatment effects for each subgroup will be produced (where significant treatment-predictor interactions are identified). For non-linear interactions, the second stage will use a multivariate meta-analysis model [75].

## Data Availability

Not applicable
